# Cochlear Neurotrophin‐3 overexpression at mid‐life prevents age‐related inner hair cell synaptopathy and slows age‐related hearing loss

**DOI:** 10.1111/acel.13708

**Published:** 2022-09-11

**Authors:** Luis R. Cassinotti, Lingchao Ji, Beatriz C. Borges, Nathan D. Cass, Aditi S. Desai, David C. Kohrman, M. Charles Liberman, Gabriel Corfas

**Affiliations:** ^1^ Kresge Hearing Research Institute and Department of Otolaryngology‐Head and Neck Surgery University of Michigan Ann Arbor Michigan USA; ^2^ Eaton‐Peabody Laboratories, Massachusetts Eye & Ear, and Department of Otolaryngology, Head and Neck Surgery Harvard Medical School Boston Massachusetts USA; ^3^ Present address: Peking University Shenzhen Hospital Shenzhen China; ^4^ Present address: The Otology Group of Vanderbilt, Vanderbilt University Medical Center Department of Otolaryngology Nashville Tennessee USA

**Keywords:** age‐related hearing loss, auditory nerve, hidden hearing loss, neurotrophins, synapse regeneration

## Abstract

Age‐related hearing loss (ARHL) is the most prevalent sensory deficit in the elderly. This progressive pathology often has psychological and medical comorbidities, including social isolation, depression, and cognitive decline. Despite ARHL's enormous societal and economic impact, no therapies to prevent or slow its progression exist. Loss of synapses between inner hair cells (IHCs) and spiral ganglion neurons (SGNs), a.k.a. IHC synaptopathy, is an early event in cochlear aging, preceding neuronal and hair cell loss. To determine if age‐related IHC synaptopathy can be prevented, and if this impacts the time‐course of ARHL, we tested the effects of cochlear overexpression of neurotrophin‐3 (Ntf3) starting at middle age. We chose *Ntf3* because this neurotrophin regulates the formation of IHC‐SGN synapses in the neonatal period. We now show that triggering Ntf3 overexpression by IHC supporting cells starting in middle age rapidly increases the amplitude of sound‐evoked neural potentials compared with age‐matched controls, indicating that Ntf3 produces a positive effect on cochlear function when the pathology is minimal. Furthermore, near the end of their lifespan, Ntf3‐overexpressing mice have milder ARHL, with larger sound‐evoked potentials along the ascending auditory pathway and reduced IHC synaptopathy compared with age‐matched controls. Our results also provide evidence that an age‐related decrease in cochlear Ntf3 expression contributes to ARHL and that Ntf3 supplementation could serve as a therapeutic for this prevalent disorder. Furthermore, these findings suggest that factors that regulate synaptogenesis during development could prevent age‐related synaptopathy in the brain, a process involved in several central nervous system degenerative disorders.

## Graphical abstract

We show that triggering cochlear Ntf3 overexpression starting in middle age rapidly increases the amplitude of sound‐evoked neural potentials and reduces age‐related inner hair cells synapse loss. Thus, near the end of their lifespan, Ntf3‐overexpressing mice have milder age‐related hearing loss and maintain larger sound‐evoked potentials along the ascending auditory pathway. These findings provide evidence that Ntf3 supplementation could serve as a therapeutic for this prevalent disorder.

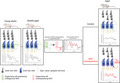

**Abbreviations**
AAV: adeno‐associated virus
ABR: auditory brainstem response
ARHL: age‐related hearing loss
CMV: cytomegalovirus
dB: decibel
DPOAE: distortion products otoacoustic emissions
HCs: hair cells
IHC: inner hair cell
Ntf3: neurotrophin‐3
OHC: outer hair cell
SGN: spiral ganglion neuron
SPL: sound pressure level
Tmx: tamoxifen

## 1 INTRODUCTION

Age‐related hearing loss (ARHL), also known as presbycusis, is the most prevalent sensory deficit in the elderly, affecting a third of people over age 65 and half of those over 85 in the US (Agrawal et al., 2008; Bainbridge & Wallhagen, 2014). This disorder is characterized by increasing progressive elevation of hearing thresholds and increasing difficulties with speech comprehension, especially in noisy environments. ARHL is also often associated with psychological and medical comorbidities, including social isolation, frailty, depression, and cognitive decline (Livingston et al., 2020; Powell et al., 2021). Despite its enormous societal and economic impact (Huddle et al., 2017), no therapies to prevent or slow ARHL exist.

While the cellular and molecular mechanisms of ARHL remain poorly understood, studies in mice and humans indicate that loss of synapses between inner hair cells (IHCs) and spiral ganglion neurons (SGNs), a.k.a. cochlear or IHC synaptopathy, is an early pathology that precedes IHC loss in mice (Sergeyenko et al., 2013) and humans (Makary et al., 2011; Viana et al., 2015; Wu et al., 2019). Furthermore, studies in mice showed that noise‐induced IHC synaptopathy at an early age accelerates the onset of SGN loss and age‐related threshold increases (Kujawa & Liberman, 2006). Based on these observations, we wondered if age‐related IHC synaptopathy can be prevented or delayed, and if achieving that alters the progression of ARHL. For this, we focused on neurotrophin‐3 (Ntf3), a neurotrophic factor critical for the survival of SGNs during embryogenesis (Ernfors et al., 1995; Farinas et al., 1994) which we showed regulates the formation of IHC‐SGN synapses in the neonatal period and induces their regeneration and functional recovery after acoustic injury in young mice (Wan et al., 2014). Moreover, using a reporter mouse in which β‐galactosidase is co‐expressed with Ntf3 (Farinas et al., 1994), we showed that cochlear Ntf3 expression decreases during aging (Sugawara et al., 2007), suggesting that lower Ntf3 levels could contribute to ARHL.

Here, we tested the impact of persistent Ntf3 overexpression starting at middle age on ARHL using transgenic mice. We show that induction of IHC supporting cell Ntf3 overexpression in 1‐year‐old mice rapidly (within 1 week) increases the amplitude of sound‐evoked cochlear potentials, something that does not occur in young mice, indicating that Ntf3 treatment acutely enhances aspects of cochlear function in middle‐aged mice with mild hearing impairment. Furthermore, Ntf3 overexpression slows the progression of ARHL, that is, as they approach the end of their lifespan (95 weeks of age), Ntf3 overexpressing mice have less age‐related IHC synaptopathy and stronger sound‐evoked potentials along the ascending auditory pathway than their age‐matched controls. Together, these results support the notion that age‐related decrease in cochlear Ntf3 expression contributes to ARHL and that Ntf3 supplementation could serve as a therapeutic for this prevalent disorder.

## 2 RESULTS

**FIGURE 1:**
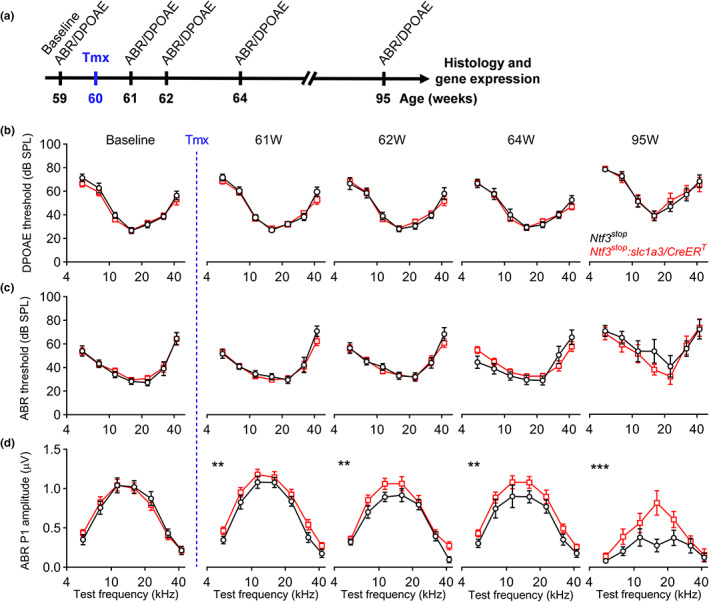
Ntf3 overexpression at mid‐life slows the age‐related decline in ABR peak 1 amplitude but does not alter age‐related ABR and DPOAE threshold shifts. (a) Timeline of the experimental design. Ntf3 overexpressing (*Ntf3* ^*stop*^ *:Slc1a3/CreER* ^*T*^) and control (*Ntf3* ^*stop*^) mice were used in this study. Ntf3 overexpression was induced by intraperitoneal tamoxifen injection at 60 weeks of age (highlighted in blue). Baseline ABRs and DPOAEs were recorded 1 week before tamoxifen treatment (59 weeks of age) and at four later time points (61, 62, 64, and 95 weeks of age). After the last ABR and DPOAE tests, cochleae were collected and processed for either immunohistochemistry followed by confocal microscopy to evaluate HCs and IHC synapse counts or for RT‐qPCR to evaluate *Ntf3* mRNA expression. (b) Ntf3 overexpression starting at 60 weeks of age does not alter DPOAE thresholds during the following 35 weeks. (c) Ntf3 overexpression starting at 60 weeks of age does not alter ABR thresholds during the following 35 weeks. (d) Ntf3 overexpression starting at 60 weeks of age increases ABR peak 1 amplitudes 1 week later and slows the decline of ABR peak 1 amplitudes at 80 dB SPL during the following 34 weeks. *Ntf3* ^*stop*^ *n* = 7–13 mice; *Ntf3* ^*stop*^ *:Slc1a3/CreER* ^*T*^ *n* = 7–17 mice. Two‐way ANOVA followed by Sidak's multiple comparisons test was used to evaluate statistical differences between *Ntf3* ^*stop*^ and *Ntf3* ^*stop*^ *:Slc1a3/CreER* ^*T*^ mice at every individual time point for either DPOAE threshold, ABR threshold or ABR peak1 amplitude. ** *p* < 0.01; *** *p* < 0.001. Error bars represent SEM

To determine the impact of increasing supporting cell Ntf3 expression on the time‐course of ARHL, we studied two cohorts of mice. One group (*Ntf3* ^*Stop*^ *:Slc1a3/CreER* ^*T*^ *)* carried an inducible *Ntf3* transgene (*Ntf3* ^*Stop*^) (Wan et al., 2014) and the *CreER* ^*T*^ recombinase transgene under the control of the GLAST (*Slc1a3*) promoter (Wang et al., 2012) to drive Ntf3 overexpression in inner border and inner phalangeal cells, the supporting cells immediately surrounding the IHCs and their synaptic connections with auditory nerve fibers. We chose this approach, because we previously showed that Ntf3 overexpression in these supporting cells promotes formation of supernumerary IHC‐SGN synapses in the neonatal period and induces synapse regeneration and functional recovery after acoustic injury in young mice (Wan et al., 2014). The control group consisted of littermates carrying only the *Ntf3* ^*Stop*^ transgene, and therefore, have normal levels of *Ntf3* expression even after tamoxifen treatment (Wan et al., 2014). At 59 weeks of age, we measured baseline auditory function in both cohorts by recording distortion product otoacoustic emissions (DPOAEs) and auditory brainstem responses (ABRs) (Figure 1a). DPOAEs reflect outer hair cell (OHC) function; ABRs reflect the function of the ascending auditory system, from the activation of IHCs and SGNs to the inferior colliculus (Kohrman et al., 2021).

**FIGURE 2:**
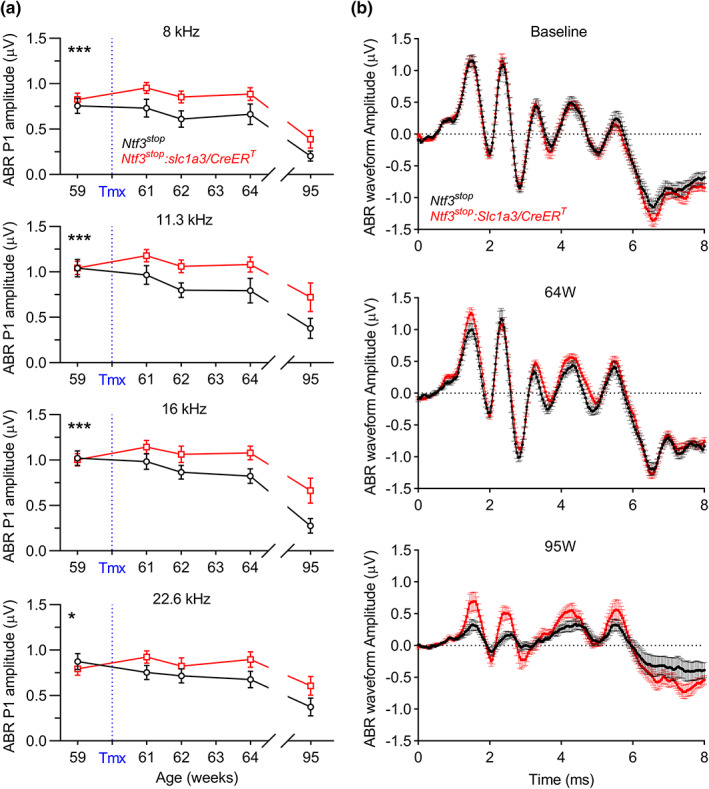
Sound‐evoked activity along the cochlear axis, and in higher auditory centers in the aging brain, is higher in aging Ntf3 overexpressing mice than in age‐matched controls. (a) After tamoxifen treatment at 60 weeks of age, ABR peak 1 amplitudes along the cochlear axis are larger in *Ntf3* ^*stop*^ *:Slc1a3/CreER* ^*T*^ mice than in *Ntf3* ^*stop*^ age‐matched controls. 2‐way ANOVA followed by Sidak's multiple comparisons test was used to evaluate ABR peak 1 amplitudes between groups over time at each individual cochlear region (frequency, kHz). (b) Mean ABR waveforms recorded at 59 (baseline), 64 and 95 weeks of age show that mice with Ntf3 overexpression have larger sound‐induced ABR peaks along the ascending auditory pathway as they age. At baseline, before tamoxifen treatment, no intergroup differences were seen in ABR waveforms (also see Figure S2). At 64 weeks, peak 1 amplitudes were increased in Ntf3 overexpressing mice. At 95 weeks, Ntf3 overexpressors exhibited smaller decreases in ABR peaks 1, 2, and 4 than *Ntf3* ^*stop*^ controls (also see Figure S2). ABRs shown here are group means in response to 16 kHz tone pips at 80 dB SPL. *Ntf3* ^*stop*^ *n* = 7–13 mice; *Ntf3* ^*stop*^ *:Slc1a3/CreER* ^*T*^ *n* = 7–17 mice. * *p* < 0.05; *** *p* < 0.001. Error bars represent SEM

The groups exhibited no differences in DPOAE and ABR thresholds at the baseline time point (59 weeks, Figure 1b,c). Furthermore, at this time point, the groups did not differ in the amplitudes of the first peak of the ABR waveform (Figures 1d and 2a), which reflects the synchronous activity of SGNs in response to sound. Analysis of the later peaks in the ABR waveforms (Figure 2b) indicated that signaling along the ascending auditory pathway at baseline was similar between the groups as well.

**FIGURE 3:**
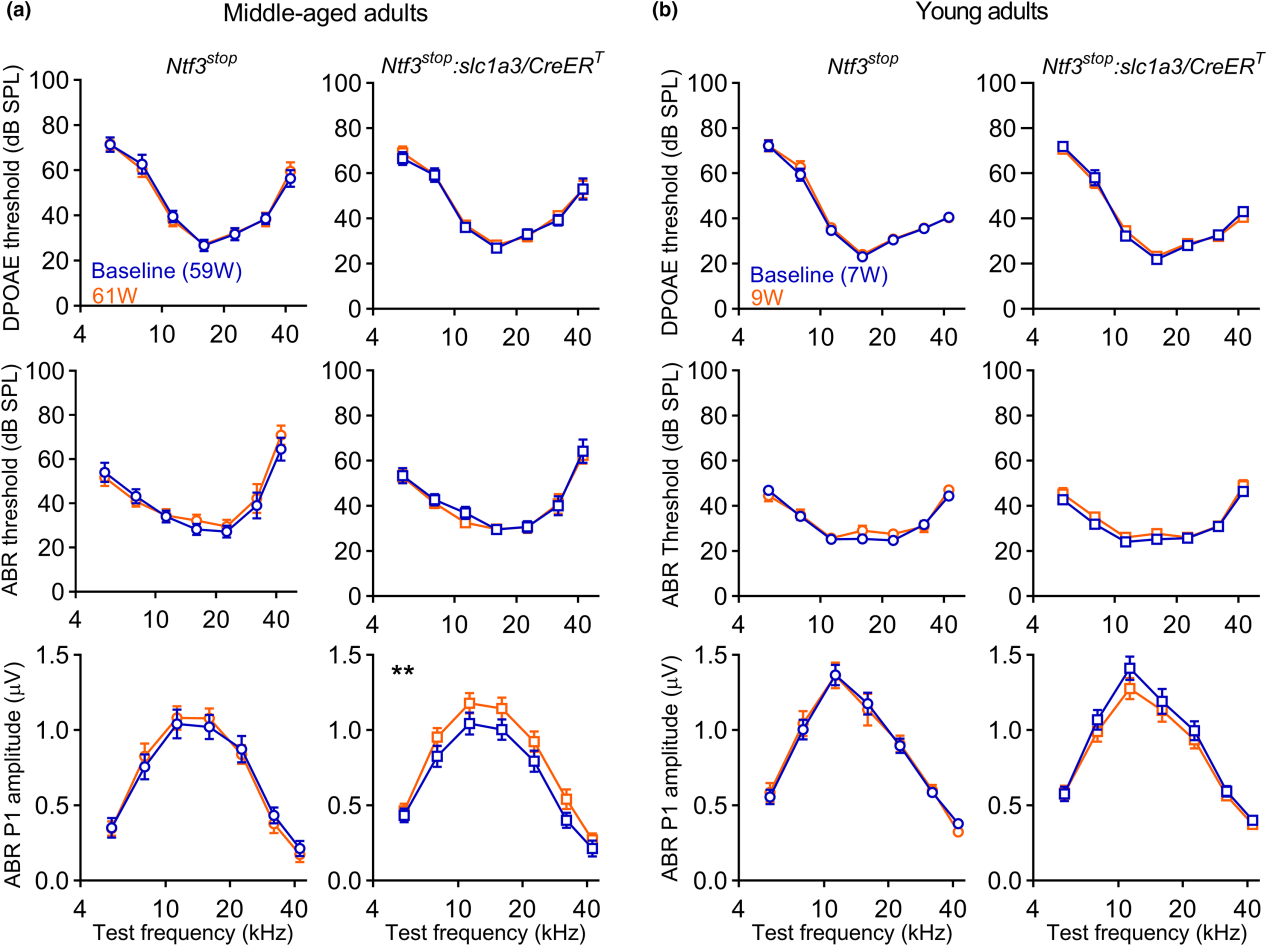
Ntf3 overexpression acutely increases ABR peak 1 amplitudes in middle‐aged mice but not in the young. (a) DPOAE and ABR recordings performed 1 week before and one week after tamoxifen injection at 60 weeks of age show that activation of Ntf3 overexpression in *Ntf3* ^*stop*^ *:Slc1a3/CreER* ^*T*^ mice rapidly increases ABR peak 1 amplitudes without altering ABR and DPOAE thresholds. This does not occur in age‐matched *Ntf3* ^*stop*^ control mice. (b) No Ntf3‐induced increases in ABR peak 1 amplitudes were seen when young adult (8‐week‐old) were tested. Suprathreshold ABR peak 1 amplitudes were analyzed at 80 dB SPL. Middle‐aged adults *Ntf3* ^*stop*^ *n* = 11 mice; Middle‐aged *Ntf3* ^*stop*^ *:Slc1a3/CreER* ^*T*^ *n* = 13–17 mice; Young adults *Ntf3* ^*stop*^ *n* = 22 mice; Young adults *Ntf3* ^*stop*^ *:Slc1a3/CreER* ^*T*^ *n* = 20 mice. 2‐way ANOVA followed by Sidak's multiple comparisons test was used to evaluate thresholds and amplitudes within each group before (baseline) and after tamoxifen treatment. ** *p* < 0.01. Error bars represent SEM

All animals were treated with tamoxifen at 60 weeks of age to induce *CreER* ^*T*^‐mediated activation of the *Ntf3* ^*Stop*^ transgene, which occurs only in mice carrying the *Slc1a3/CreER* ^*T*^ allele, and auditory function was followed longitudinally in both cohorts. Notably, 1 week after activation of Ntf3 overexpression (61 weeks of age), the suprathreshold ABR peak 1 amplitudes in *Ntf3* ^*Stop*^ *:Slc1a3/CreER* ^*T*^ mice were higher than in age‐matched controls (Figures 1d, 2a and 3a), indicating that increased Ntf3 availability leads to an acute increase in the sound‐evoked potentials mediated by IHC‐SGN synapses. Interestingly, activation of the *Ntf3* transgene did not alter ABRs and DPOAE thresholds or ABR1 peak 1 amplitudes in young (8‐week‐old) mice (Figure 3b). Since by 59 weeks, the mice already exhibit a mild but significant reduction in ABR peak 1 amplitudes (Figure S1), and cochlear Ntf3 expression steadily decreases with age (Sugawara et al., 2007), these data suggest that Ntf3 overexpression can have acute effects on ABR peak 1 amplitudes only when Ntf3 endogenous levels are reduced.

As the animals aged, both groups showed similar increases in DPOAE and ABR thresholds (Figure 1b,c), indicative of ARHL. However, suprathreshold ABR peak 1 amplitudes were higher in *Ntf3* ^*Stop*^ *:Slc1a3/CreER* ^*T*^ than in the age‐matched *Ntf3* ^*Stop*^ controls at all post‐tamoxifen time points (Figure 1d) and all test frequencies (Figure 2a), indicating that Ntf3 overexpression preserves the number and/or strength of connections between IHCs and SGNs during aging, all along the cochlear axis. Importantly, Ntf3 overexpression also enhanced most of the later peaks in the ABR waveforms at 95 weeks of age (Figure 2b and Figure S2), showing that the Ntf3‐mediated increase in ABR peak 1 amplitudes is also reflected in higher synchronous activation along the ascending auditory pathway in the aging brain.

**FIGURE 4:**
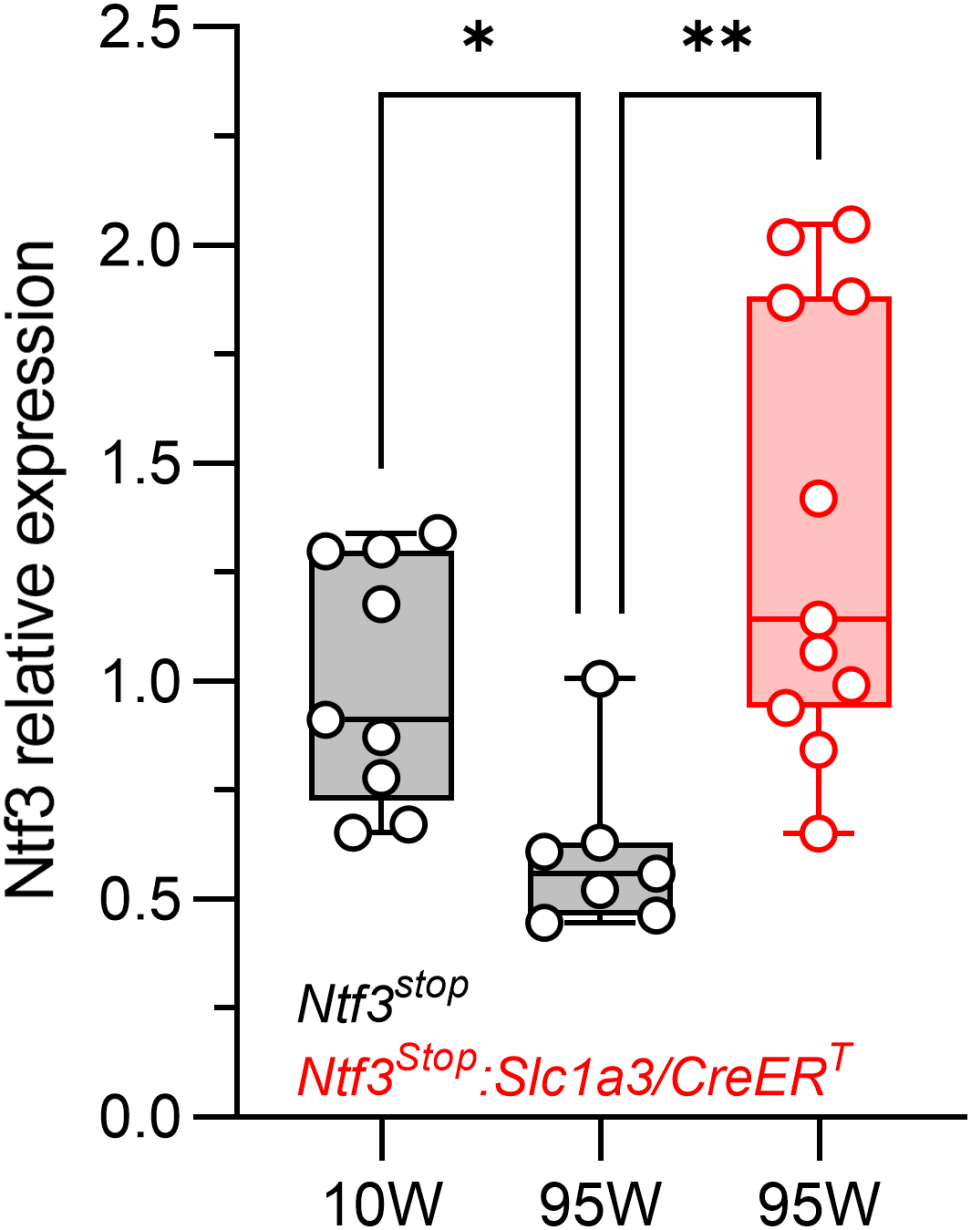
Cochlear *Ntf3* mRNA levels decrease by 40% between 10 and 95 weeks of age in control mice, but expression levels in 95‐week‐old *Ntf3* ^*stop*^ *:Slc1a3/CreER* ^*T*^ mice remain the same as in young mice when tamoxifen is injected at 60 weeks of age. *Ntf3* mRNA levels in cochleae from control (*Ntf3* ^*stop*^) mice at 10 and 95 weeks of age (grey bars) and in 95‐week‐old *Ntf3* ^*stop*^ *:Slc1a3/CreER* ^*T*^ mice that were treated with tamoxifen at 60 weeks. 10‐week‐old *Ntf3* ^*stop*^ *n* = 9 cochleae; 95‐week‐old *Ntf3* ^*stop*^ *n* = 7 cochleae; 95‐week‐old *Ntf3* ^*stop*^ *:Slc1a3/CreER* ^*T*^ *n* = 11 cochleae (only one cochlea per mouse was analyzed). Kruskal–Wallis test, followed by Dunn's multiple comparisons test was used to evaluate differences among the three groups. * *p* < 0.05; ** *p* < 0.01. Error bars represent SEM

After the last session of auditory testing, inner ears were harvested and used for molecular and histological analysis. *Ntf3* mRNA levels were significantly higher in the 95‐week‐old *Ntf3* ^*Stop*^ *:Slc1a3/CreER* ^*T*^ mice than in their 95‐week‐old littermates carrying only the *Ntf3* ^*Stop*^ transgene (Figure 4), indicating that we had achieved persistent transgenic expression. Furthermore, consistent with our prior studies (Sugawara et al., 2007), *Ntf3* levels in control mice at 95 weeks were ~ 40% lower than those in 10‐week‐old controls. Notably, *Ntf3* levels in *Ntf3* ^*Stop*^ *:Slc1a3/CreER* ^*T*^ mice at 95 weeks were not statistically different from those seen in young control (*Ntf3* ^*Stop*^) mice, indicating that tamoxifen induction was sufficient to maintain *Ntf3* expression in the old *Ntf3* ^*Stop*^ *:Slc1a3/CreER* ^*T*^ mice at the same level as they had in their youth.

**FIGURE 5:**
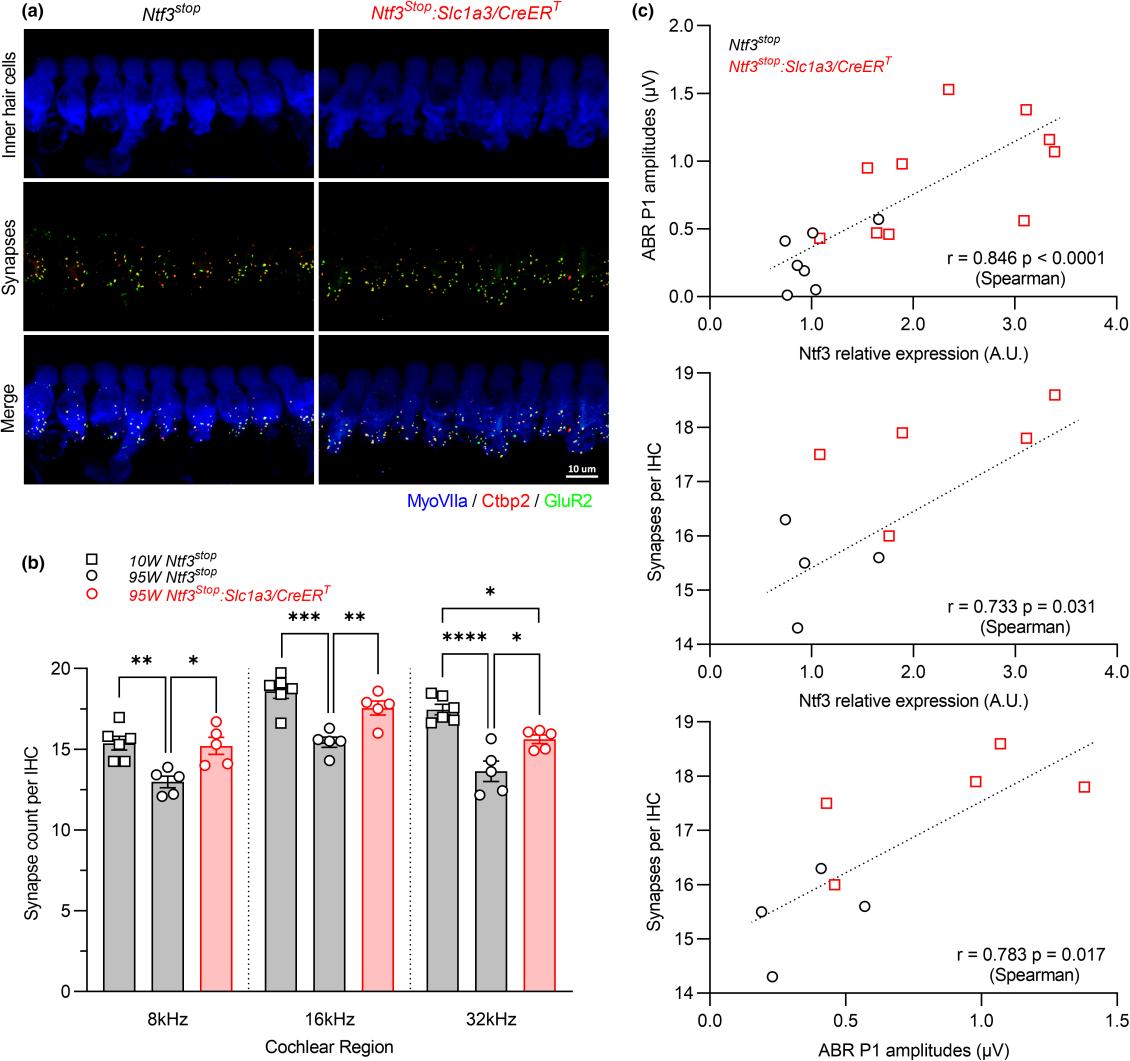
Induction of *Ntf3* expression starting at 60 weeks of age results in increased IHC synapse densities and ABR peak 1 amplitudes at 95 weeks of age. (a) Representative confocal images of IHC synapses at the 16 kHz region of a 95‐week‐old *Ntf3* ^*stop*^ and a *Ntf3* ^*stop*^ *:Slc1a3/CreER* ^*T*^ cochlea immunolabeled for hair cells (MyoVIIa), pre‐synaptic ribbons (Ctbp2) and post‐synaptic receptor patches (GluR2). (b) At 95 weeks of age, Ntf3 overexpressors (*Ntf3* ^*stop*^ *:Slc1a3/CreER* ^*T*^) have 14–17% more IHC synapses than their age‐matched controls (*Ntf3* ^*stop*^), a density not different than that seen in 10‐week‐old controls. 10‐week‐old *Ntf3* ^*stop*^ *n* = 6 cochleae; 95‐week‐old *Ntf3* ^*stop*^ *n* = 5 cochleae; 95‐week‐old *Ntf3* ^*stop*^ *:Slc1a3/CreER* ^*T*^ *n* = 5 cochleae. Only one cochlea from each mouse was analyzed. 1‐way ANOVA followed by Tukey's multiple comparisons test was used to evaluate differences among the three groups at an individual cochlear region (frequency, kHz). * *p* < 0.05; ** *p* < 0.01; *** *p* < 0.001; **** *p* < 0.0001 by one‐way ANOVA. Error bars represent SEM. (c) *Ntf3* expression levels, ABR peak 1 amplitudes, and IHC synapses density strongly correlate in 95‐week‐old mice. Correlations were evaluated using Spearman correlation test. *p* < 0.05 were considered as statistically significant differences

Consistent with the preservation of ABR peak 1 amplitudes in 95‐week‐old *Ntf3* ^*Stop*^ *:Slc1a3/CreER* ^*T*^ mice, their IHC‐SGN synapse counts were also similar to those found in 10‐week‐old *Ntf3* ^*Stop*^ mice, and significantly higher than in the 95‐week‐old *Ntf3* ^*Stop*^ littermates (Figure 5a,5b), demonstrating that Ntf3 overexpression prevented age‐related IHC synaptopathy. Furthermore, we found a significant correlation among *Ntf3* mRNA levels, synapse counts and ABR peak 1 amplitudes at 95 weeks of age (Figure 5c; ABR peak 1 amplitudes vs. *Ntf3* expression level *r* = 0.846 *p* < 0.0001; IHC synapses vs. *Ntf3* expression level *r* = 0.733 *p* = 0.031 and IHC synapses vs. ABR peak 1 amplitudes *r* = 0.783 *p* = 0.017).

**FIGURE 6:**
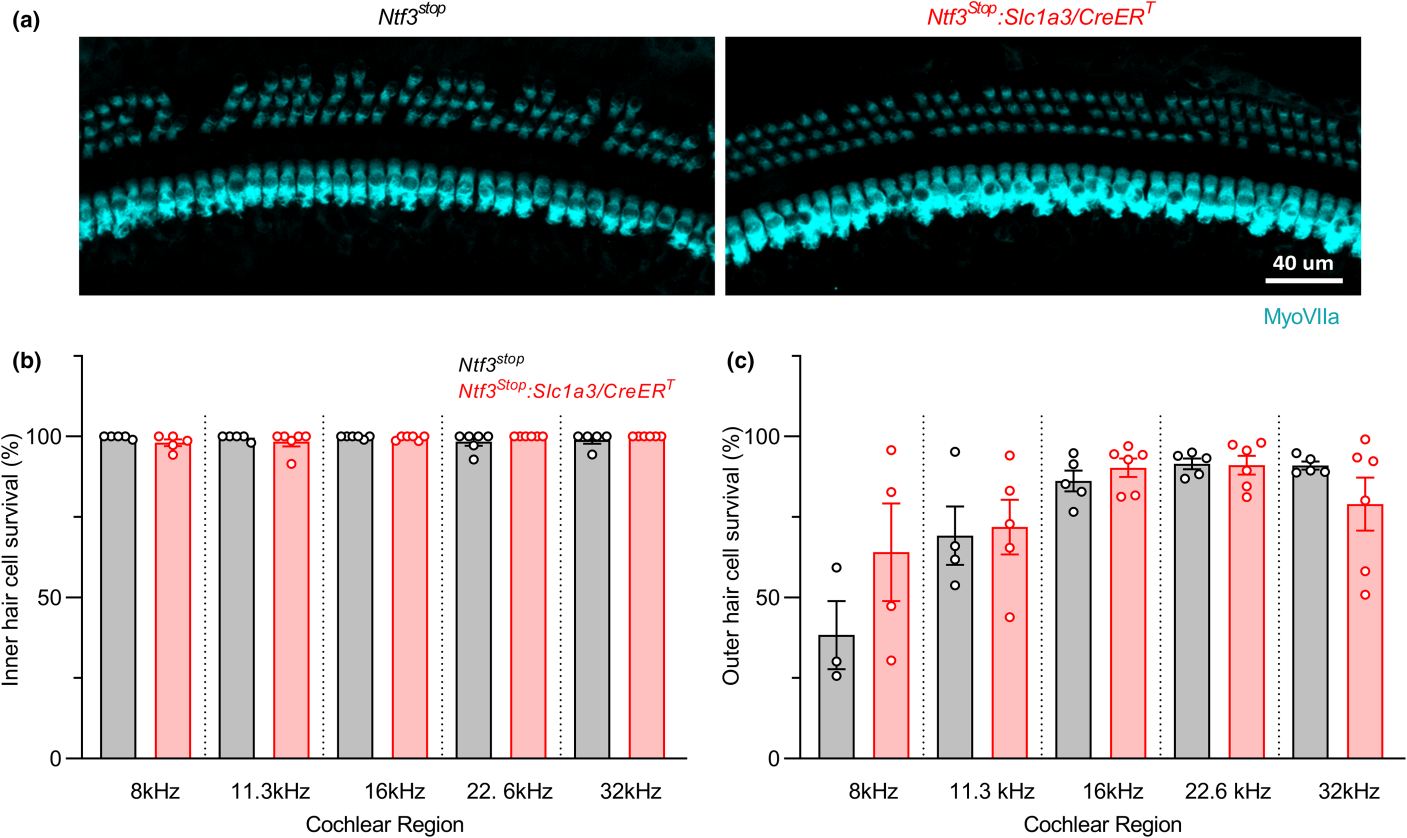
Ntf3 overexpression starting at mid‐life does not alter age‐related OHC loss. (a) Representative confocal images of the 16 kHz region of *Ntf3* ^*stop*^ and *Ntf3* ^*stop*^ *:Slc1a3/CreER* ^*T*^ cochleae immunolabeled for hair cells (MyoVIIa) showing mild OHC loss without IHC loss in both groups at 95 weeks of age. (b) Quantitative analysis shows no IHC loss. (c) Both groups show equal degrees of mild OHC loss. *Ntf3* ^*stop*^ *n* = 3–6 cochleae*; Ntf3* ^*stop*^ *:Slc1a3/CreER* ^*T*^ *n* = 5–6 cochleae. Only one cochlea from each mouse was analyzed. 2‐tailed t‐test was used to evaluate differences between *Ntf3* ^*stop*^ and *Ntf3* ^*stop*^ *:Slc1a3/CreER* ^*T*^ groups at an individual cochlear region (frequency, kHz). Non‐significant differences between groups were found when evaluating either ICH or OHC survival. Error bars represent SEM

In contrast, in agreement with the DPOAE thresholds shifts seen in both genotypes at 95 weeks (Figure 1b), *Ntf3* ^*Stop*^ *:Slc1a3/CreER* ^*T*^ and *Ntf3* ^*Stop*^ mice had similar levels of OHC loss at that age (Figure 6c). This indicates that supporting cell Ntf3 overexpression protects IHC‐SGN synapses but not OHCs, which do not express the receptor for Ntf3 (TrkC) and only transiently express the receptor for Bdnf (TrkB) during the neonatal period (Knipper et al., 1996; Ylikoski et al., 1993).

## 3 DISCUSSION

ARHL has reached epidemic proportions (Agrawal et al., 2008; Bainbridge & Wallhagen, 2014), and hearing impairment has been shown to contribute to cognitive decline and dementia (Livingston et al., 2020; Powell et al., 2021). Thus, developing therapies to prevent ARHL is an important public health priority. Our results provide the first demonstration that increasing Ntf3 levels in the middle‐aged cochlea promotes the maintenance of IHC‐SGN synapses and their function during murine aging, supporting the potential of Ntf3 supplementation as a potential strategy to reduce the burden of ARHL in humans. However, the increased Ntf3 expression and IHC synapse preservation does not prevent OHC loss and the consequent age‐related cochlear threshold shifts. These findings indicate that OHC survival during aging does not depend on Ntf3, SGNs, or IHC‐SGN synapses, and that strategies to enhance OHC survival will be necessary to complement the potential therapeutic effects of Ntf3. Importantly, despite the threshold shifts observed in the aged Ntf3‐overexpressing mice, amplitudes of most peaks of the ABR waveform were larger than in their age‐matched controls, suggesting that the Ntf3‐induced preservation of IHC‐SGN synapses and ABR peak 1 amplitudes improves signaling along the ascending auditory pathway during aging.

Whereas good cochlear thresholds are key to maintaining the audibility of sounds, healthy neural populations are key to maintaining the intelligibility of sounds, especially in difficult listening situations, where the central nervous system likely pools responses from large population of auditory neurons to improve the signal‐to‐noise ratio (Lopez‐Poveda, 2014). Histopathological studies of human autopsy specimens have demonstrated a dramatic loss of auditory nerve fibers in “normal‐aging” adults, despite the survival of most of their IHC targets (Wu et al., 2019). It is likely that this IHC‐SGN synapse loss and primary neural degeneration are key contributors to the classic complaint of those with ARHL, that is, problems understanding speech in a noisy environment (Lopez‐Poveda, 2014). The present results suggest that Ntf3 therapies could prevent or slow much of this age‐related degradation in hearing abilities.

We previously showed that IHC‐SGN synapse density and ABR peak 1 amplitude strongly correlate in young mice (Wan et al., 2014). Here, we demonstrate that these variables also correlate with Ntf3 expression levels in aged mice. Our results also show that activating Ntf3 overexpression in the middle‐aged cochlea rapidly increases the amplitude of cochlear sound‐evoked potentials without altering ABR or DPOAE thresholds. Remarkably, this does not occur when Ntf3 is overexpressed in young adults (2‐month‐old). This difference likely reflect the age‐related decline in endogenous Ntf3 expression (Figure 4 and (Sugawara et al., 2007), that is, Ntf3 overexpression enhances IHC‐SGN synapse numbers and/or function acutely only after endogenous Ntf3 expression is reduced due to aging. Together with the finding that Ntf3 overexpression in middle age prevents subsequent loss of IHC‐SGN synapses, our results suggest that the age‐related decline in endogenous Ntf3 expression contributes to age‐related IHC synaptopathy and reductions in ABR peak 1 amplitudes.

Our previous studies on noise‐induced synaptopathy showed that increasing cochlear Ntf3 levels promotes synapse preservation or regeneration when the neurotrophin is provided via transgenically driven overexpression (Wan et al., 2014), by direct Ntf3 application to the round window (Suzuki et al., 2016) or by AAV‐mediated gene transfer (Hashimoto et al., 2019). Thus, we anticipate that both protein delivery and gene therapy, in combination with new approaches for Ntf3 delivery to the round window (Gunewardene et al., 2022; Wang et al., 2021) could also work for ARHL. Of course, a virus‐mediated gene therapy approach would have the advantage of long‐lasting expression that is likely necessary to preserve IHC‐SGN synapses over years, similar to the effects we observed here in transgenic mice. However, other studies indicate that careful control of virus titer and levels of Ntf3 overexpression will be necessary to avoid negative side effects. For example, we found that while relatively modest levels of Ntf3 overexpression (2–4‐fold over control) via AAV‐mediated gene therapy (Hashimoto et al., 2019) or transgenesis (Wan et al., 2014) promote recovery of IHC‐SGN synapses after noise exposure in young mice, higher Ntf3 overexpression levels in IHCs (50–100‐fold over control) can increase IHC death after noise exposure (Hashimoto et al., 2019).

Synaptopathy is not only involved in the pathogenesis of ARHL but has been proposed to play early and critical roles in a number of neurological and neurodegenerative disorders (Henstridge et al., 2016). Thus, our findings in the cochlea raise the possibility that increasing the availability of factors that regulate synaptogenesis in the central nervous system during development might serve as therapies for degenerative disorders by slowing or preventing age‐related synaptopathy.

## 4 EXPERIMENTAL PROCEDURES

### 4.1 Animals and tamoxifen treatment

To overexpress Ntf3 from adult supporting cells, we crossed *Slc1a3/CreER* ^*T*^ mice (*Slc1a3 CreER* ^*T*^; JAX stock no. 012586) with *Ntf3* ^*stop*^ mice. The conditional *Ntf3* overexpression transgene is under regulation of a strong ubiquitous promoter (CAGGS), which contains the cytomegalovirus (CMV) early enhancer element; the promoter, first exon and first intron of the chicken beta‐actin gene; and the splice acceptor of the rabbit beta‐globin gene. Between the CAGGS and the Ntf3 sequences, we included a floxed STOP cassette that prevents transgene expression until it is removed by the Cre recombinase (regular or tamoxifen inducible). Unless epigenetic mechanisms silence the transgene (which can occur), transgenic expression tends to be long‐lasting.

*Slc1a3/CreER* ^*T*^ mice (Wang et al., 2012) were obtained from Jackson Laboratory while the engineering of the *Ntf3* ^*stop*^ mouse was described elsewhere (Wan et al., 2014). *Ntf3* ^*stop*^ *:Slc1a3/CreER* ^*T*^ mice and their controls were on a mixed background of C57BL/6 and FVB/N, and were genotyped to ensure that they do not carry the *ahl* cadherin 23 allele associated with early onset progressive hearing loss present in C57BL/6 mice (Noben‐Trauth et al., 2003). Cre‐negative littermates (*Ntf3* ^*stop*^) were used as control group. For the study, either 8‐week‐old or 60‐week‐old mice were gavaged with tamoxifen (200 mg/kg/day) for 3 days. Mouse ages were chosen following criteria correlating maturational rates of mice and humans (Flurey et al., 2007; Hagan, 2017; The‐Harrison‐lab, n.d). Since the two are not linearly correlated, these authors consider 3–6 month‐old mice as mature adults, 10–15 month‐old mice as middle‐aged, and 18–24 month‐old as old. Therefore, we used 8‐week‐old mice as young, 60‐week‐old as middle‐aged, and 95‐week‐old as old mice. All animal procedures were approved by Institutional Animal Care and Use Committee of the University of Michigan, and all experiments were performed in accordance with relevant guidelines and regulations.

### 4.2 Physiological analyses

Inner ear physiology, including auditory brainstem responses (ABRs, the summed activity of auditory afferent pathways to short tone bursts), and distortion product otoacoustic emissions (DPOAEs), was performed on mice anesthetized with a mixture of ketamine (100 mg/kg, i.p.) and xylazine (20 mg/kg, i.p.). For the middle‐aged mice, the first recording was performed at 59 weeks of age (baseline), followed by tamoxifen treatment at 60 weeks of age and additional measurements at 61, 62, 64, and 95 weeks of age. For the young adult mice, the first recording was performed at 7 weeks of age (baseline), followed by tamoxifen treatment at 8 weeks of age and additional measurement at 9 weeks of age. For ABR recordings, acoustic stimuli were delivered through a closed acoustic system, consisting of two sound sources (CDMG15008‐ 03A, CUI) and an electret condenser microphone (FG‐23329‐PO7, Knowles) as an in‐dwelling probe microphone. Three needle electrodes were placed into the skin at the dorsal midline: one close to the neural crest, one behind the left pinna, and one at the base of the tail (ground). ABR potentials were evoked with 5 ms tone pips (0.5 ms rise‐fall, with a cos^2^ envelope, at 40/s) delivered to the eardrum at log‐spaced frequencies from 5.6 to 42.25 kHz. The response was amplified (10,000X) and filtered (0.3–3 kHz) with an analog‐to‐digital board in a PC‐based data‐acquisition system. Sound pressure level was raised in 5 dB steps from 20 to 80 dB SPL. At each level, 1024 responses were averaged (with stimulus polarity alternated) after “artifact rejection” above 15 μV. The DPOAEs, in response to two primary tones of frequencies f1 and f2, were recorded at (2 × f1) − f2, with f2/f1 = 1.2, and the f2 level 10 dB lower than the f1 level. Stimuli were raised in 5 dB steps from 20 to 80 dB. The ear‐canal sound pressure was amplified and digitally sampled at 4 μs intervals. DPOAE thresholds were defined as the lower SPL where (2f1‐f2) ‐ (2f1‐f2Nse) > 0. These acoustic signals, generated by outer hair cells and measurable in the ear canal, are useful for differential diagnosis: attenuation of ABRs without a change in DPOAEs provides strong evidence for IHC‐SGN synaptic or neural dysfunction (Kujawa & Liberman, 2009). Both ABR and DPOAE recordings were performed using the EPL Cochlear Function Test Suite (Mass Eye and Ear, Boston,). ABR thresholds, ABR peak 1 amplitudes and latencies, ABR waveforms and DPOAE thresholds were analyzed with ABR peak Analysis software (Mass Eye and Ear, Boston,) and Microsoft Excel.

### 4.3 RNA isolation and quantitative RT‐PCR

Mice (10‐ and 95‐weeks‐old) were euthanized by cervical dislocation followed by decapitation and the inner ear temporal bones were harvested. Total RNA was purified from whole individual inner ears using RNA extraction kit and Qiazol Reagent (RNeasy mini kit; Qiagen,). DNase treatment was performed immediately after total RNA purification (RNase‐free; Qiagen). First strand cDNA was prepared from 400 ng of total RNA and the complementary DNA strand was synthesized using iScript cDNA synthesis kit (Bio‐Rad, #1708891), according to the manufacturers protocol. Quantitative RT‐PCR was carried out by triplicate using cDNA aliquots derived from each of 9 inner ears (10‐week‐old *Ntf3* ^*stop*^), 7 inner ears (95‐week‐old *Ntf3* ^*stop*^), and 11 inner ears (95‐week‐old *Ntf3* ^*stop*^ *:Slc1a3/CreER* ^*T*^). The 10 μl reaction contained 5 μl of SYBR Green supermix, 6 pmol of each forward and reverse primer (0.6 μl), 1.9 μl nuclease‐free of water and 2.5 μl of cDNA sample. cDNA samples were amplified using iTaq Universal SYBR® Green supermix (Bio‐Rad, # 172–5121). Upon PCR completion, PCR products were melted by gradually increasing the temperature in 0.5°C steps. Each sample had a matched “no‐RT” control which was tested simultaneously. Water instead of complementary DNA was used as a negative control. RT‐qPCR was performed on a CFX‐96 Bio‐Rad reverse transcription polymerase chain reaction detection system (Hercules,). Relative transcript levels of the *Ntf3* gene were determined by a comparative cycle threshold (Ct) method and relative gene copy number was calculated as normalized gene expression, defined as described previously (Stankovic & Corfas, 2003). Changes in mRNA expression were calculated as relative expression (arbitrary units) respective to the control group. Ribosomal protein L19 (*Rpl19*) was used as the housekeeping gene for normalization. Primers pairs were synthesized by IDT (Coralville, IA,): *Ntf3*, F 5' GCCCCCTCCCTTATACCTAATG 3'; R: 5' CATAGCGTTTCCTCCGTGGT 3'; *Rpl19*, F: 5' ACCTGGATGAGAAGGATGAG 3'; R: 5' ACCTTCAGGTACAGGCTGTG 3'.

### 4.4 Immunostaining for hair cells and synaptic counts

Inner ear tissues from 10‐ and 95‐week‐old mice were dissected and fixed in 4% paraformaldehyde in 0.01 M phosphate‐buffered saline (PBS) for 2 h at room temperature, followed by decalcification in 5% EDTA at 4°C for 5 days. Cochlear tissues were microdissected and permeabilized by freeze–thawing in 30% sucrose. The microdissected tissues were incubated in blocking buffer containing 5% normal goat serum and 0.3% Triton X‐100 in PBS for 1 h. Tissues were then incubated in primary antibodies (diluted in 1% normal goat serum and 0.3% Triton X‐100 in PBS) at 37°C overnight. The primary antibodies used in this study were as follows: anti‐Ctbp2 (BD Biosciences, San Jose, CA; 1:200; catalog no. 612044), anti‐GluR2 (Millipore, Billerica, MA; 1:1000; catalog no. MAB397), and anti‐MyoVIIa (Proteus Biosciences, Ramona, CA; 1:100; catalog no. 25–6790). Tissues were then incubated with appropriate Alexa Fluor‐conjugated fluorescent secondary antibodies (Invitrogen, Carlsbad, CA; 1:1000 diluted in 1% normal goat serum and 0.3% Triton X‐100 in PBS; AF488 IgG2a catalog no. A‐21131; AF568 IgG1 catalog no. A‐21124; AF647 IgG catalog no. A‐21244) for 1 h at room temperature. The tissues were mounted on microscope slides in ProLong Diamond Antifade Mountant (Thermo Fisher Scientific). All pieces of each cochlea were imaged at low power (10X magnification) to convert cochlear location into frequency (tonotopic mapping) using a custom plug‐in to ImageJ (1.53c NIH, MD) available at the website of the Eaton‐Peabody Laboratories (EPL). Cochlear tissues from 8 to 32 kHz regions were used for further analyses. Confocal z‐stacks of cochlear tissues were taken using a Leica SP8 confocal microscope. Images for hair cell counts were taken under 40X magnification. For inner hair cell synapse counts, z‐stacks (0.3 μm step size) were taken under 63X (+2.4X optical zoom) magnification spanning the entire IHC height to ensure all synapses were imaged. Imaging and analyses of cochlear hair cells and synapses were performed as previously described in (Wan et al., 2014). Briefly, imageJ/Fiji software (version 1.53c, NIH, MD) was used for image processing and quantification. One cochlea from each of five or six animals was imaged for each experiment, with 3 images acquired at each cochlear region. For hair cell quantification, the number of inner and outer hair cells at specific cochlear regions in each animal was determined based on the MyoVIIa channel and counted manually using ImageJ/Fiji software multi‐point counter tool. For synapse counts, CtBP2 and GluR2 puncta in each image stacks were also captured and counted manually using ImageJ/Fiji software multi‐point counter tool. Synaptic counts of each z‐stack were divided by the number of IHCs, which could be visualized by staining of MyoVIIa antibody. Each individual image usually contained 8–10 IHCs. For figures, one representative image was selected from amongst the 15–18 images from the specific frequency shown.

### 4.5 Statistical analysis and correlations

Graphics and statistical tests were performed using GraphPad Prism version 9.3.1 for Windows (GraphPad Software, www.graphpad.com). Data sets with normal distributions were analyzed with parametric tests whereas non‐parametric tests were used for sets that did not conform to normality criteria. We used 2‐way ANOVA, followed by Sidak's multiple comparisons test to compare DPOAE thresholds, ABR thresholds, and ABR peak1 amplitudes at each time point (59, 60, 62, 64 and 95 weeks) between *Ntf3* ^*stop*^ *:Slc1a3/CreER* ^*T*^ and *Ntf3* ^*stop*^ (Figures 1b,c). Two‐way ANOVA followed by Sidak's multiple comparisons test was also used to evaluate ABR peak 1 amplitudes between groups over time at an individual cochlear region (frequency, kHz) (Figure 2a). Two‐way ANOVA followed by Sidak's multiple comparisons test was used to evaluate the acute effect of Ntf3 overexpression on DPOAE thresholds, ABR thresholds, and ABR peak1 amplitudes (Figure 3) within each genotype. *Ntf3* mRNA expression levels were analyzed using Kruskal–Wallis test followed by Dunn's multiple comparisons test. Quantification of confocal microcopy images were analyzed by two‐tailed t‐test for inner and outer hair cells survival, and 1‐way ANOVA followed by Tukey's multiple comparisons test for IHC‐SGNs synapse density. Correlations were evaluated using Spearman correlation test. All *p* < 0.05 were considered as statistically significant.

## AUTHOR CONTRIBUTIONS

LRC and GC designed the research studies. LRC, LJ, BCB, and NDC conducted experiments. LRC, LJ, BCB, NDC, ASD, and DCK acquired data. LRC, BCB, ASD, and GC analyzed data. LRC, LJ, BCB, NDC, ASD, DCK, MCL, and GC wrote the manuscript.

## CONFLICT OF INTEREST

GC and MCL are scientific founders of Decibel Therapeutics, have equity interest in the company and have received compensation for consulting. The company was not involved in this study.

## Supporting information


Figure S1–S2

## Data Availability

The data that support the findings of this study are available from the corresponding author upon reasonable request.
