# Supplementary material for: Cochlear Neurotrophin‐3 overexpression at mid‐life prevents age‐related inner hair cell synaptopathy and slows age‐related hearing loss
Source: Aging Cell. 2022 Sep 11;21(10):e13708. doi: 10.1111/acel.13708 (PMC9577954; doi:10.1111/acel.13708)
Supplement: Supplementary file 1 — Figure S1–S2 [file ACEL-21-e13708-s001.docx]

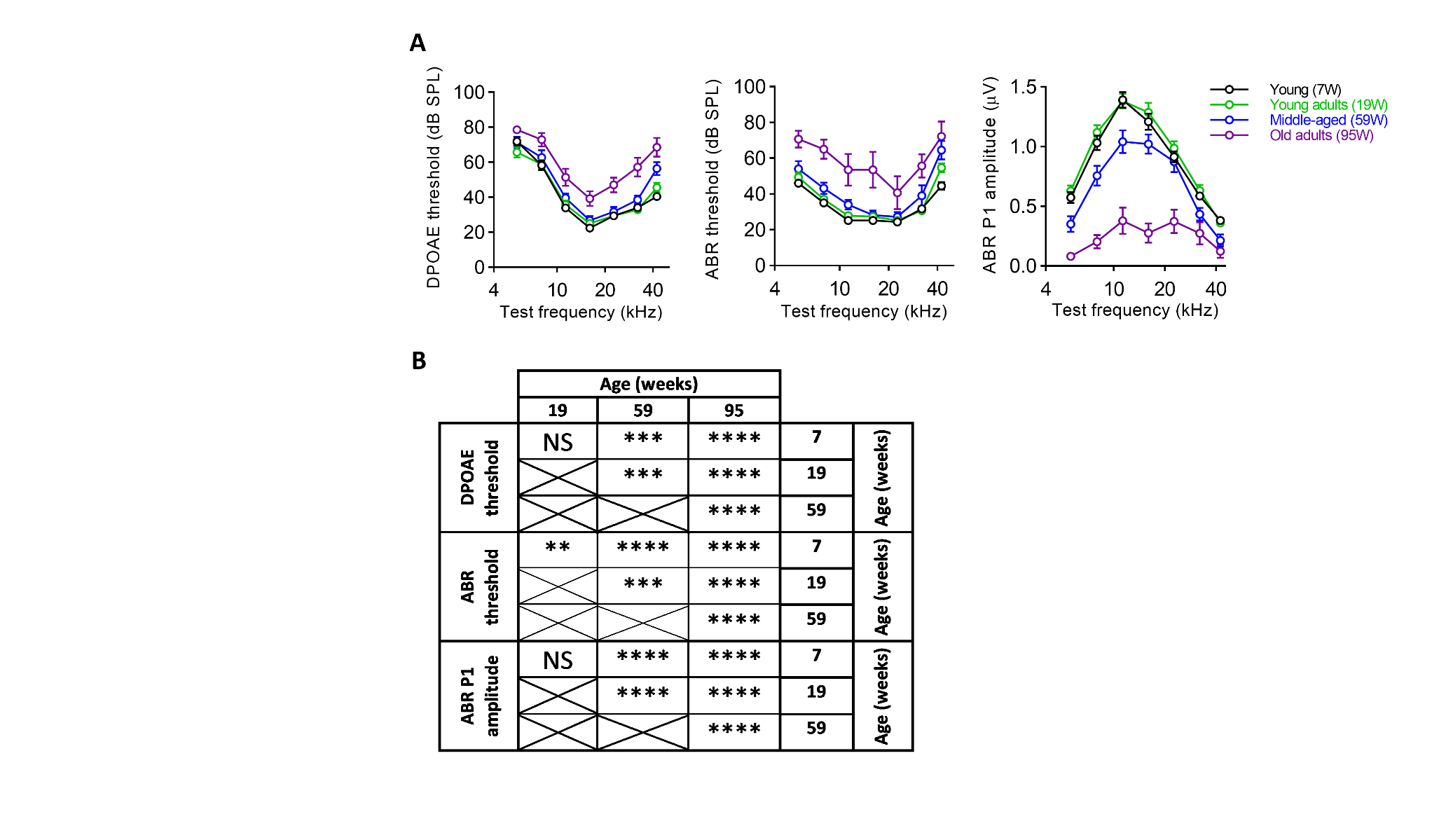


**Supplementary figure 1. Progression of age-related hearing loss in control mice (*Ntf3^stop^*)**. **(A)** DPOAE threshold, ABR threshold, and ABR peak 1 amplitude at 80 dB sound pressure level (SPL) measured on 7- (n = 23), 19- (n = 19), 59- (n = 11) and 95- (n = 7) week-old control mice. Error bars represent SEM. **(B)** Two-way ANOVA results comparing 7-, 19-, 59- and 95-week-old control mice. DPOAE threshold, ABR threshold and ABR peak 1 amplitude were measured at these ages. ** p < 0.01, ***p < 0.001 and ****p < 0.0001.


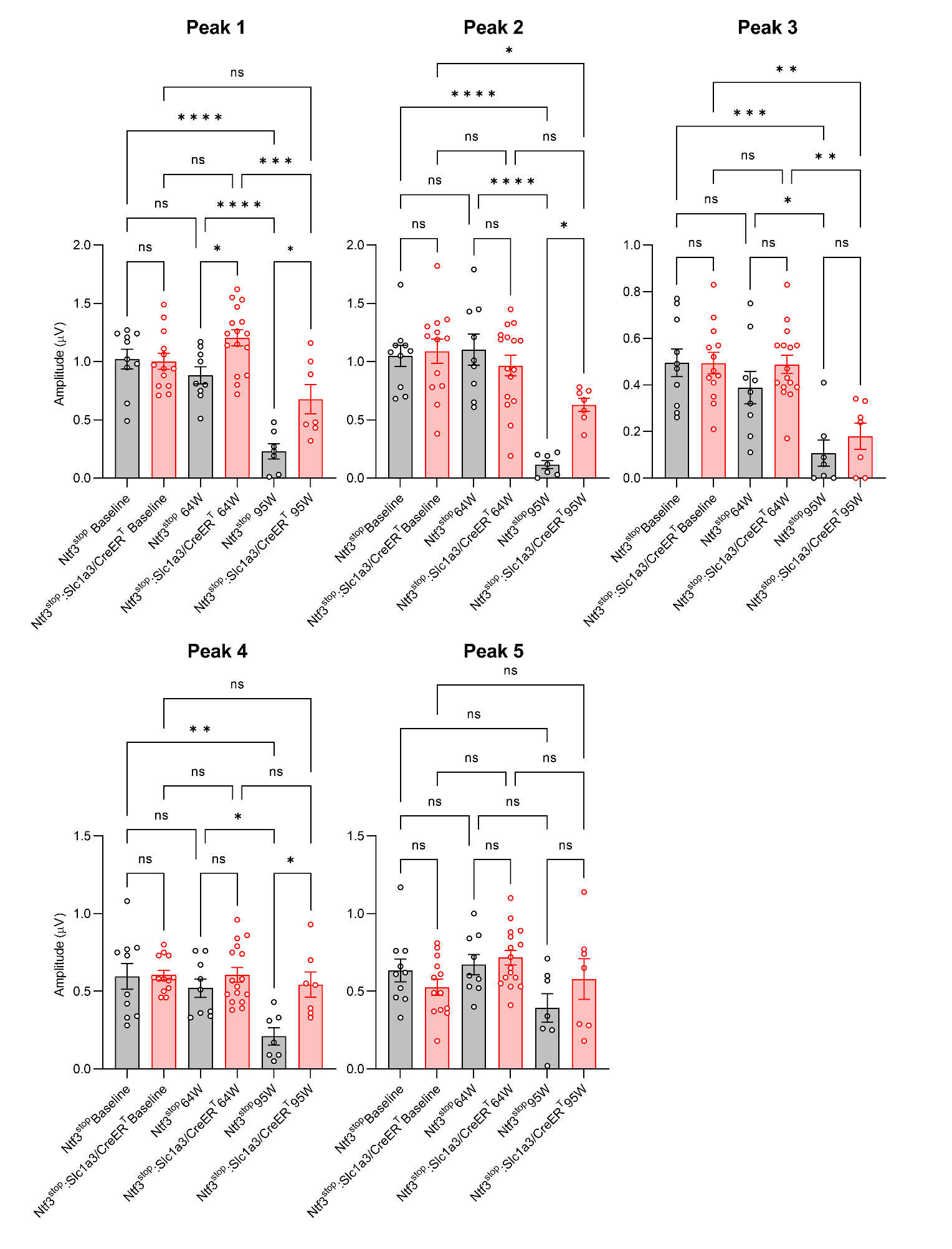


**Supplementary figure 2.** **Ntf3 overexpression at mid-life slows the age-related decline in ABR peaks 1, 2 and 4.** Four weeks after tamoxifen induced Ntf3 overexpression (64 wks), ABR peak 1 amplitudes are larger in *Ntf3^stop^:Slc1a3/CreER^T^* mice compared with age-matched controls. ABR peak 1 amplitudes remain larger in the overexpressing mice as the animal at 95 weeks of age. ABR peak 2 and 4 amplitudes are also larger in 95-week-old *Ntf3^stop^:Slc1a3/CreER^T^* mice compared with age-matched controls. ABR peak 3 and 5 amplitudes are not altered by Ntf3 overexpression at mid-life, most likely because their magnitudes are smaller. ABR peak amplitudes were analyzed at 80 dB sound pressure level (SPL). Statistical differences were evaluated by one-way ANOVA followed by Tukey’s multiple comparison test. * p < 0.05, ** p < 0.01, ***p < 0.001 and ****p < 0.0001. Error bars represent SEM.
